# Evidence of weak Anderson localization revealed by the resistivity, transverse magnetoresistance and Hall effect measured on thin Cu films deposited on mica

**DOI:** 10.1038/s41598-021-97210-w

**Published:** 2021-09-08

**Authors:** Eva Díaz, Guillermo Herrera, Simón Oyarzún, Raul C. Munoz

**Affiliations:** 1 Departamento de Física, Facultad de Ciencias Físicas Y Matemáticas, Universidad de Chile Blanco Encalada 2008, Casilla 487-3, 8370449 Santiago, Chile; 2 Departamento de Física, CEDENNA, Universidad de Santiago de Chile, USACH, Av. Ecuador 3493, 9170124 Santiago, Chile

**Keywords:** Applied physics, Nanoscale materials, Electronic properties and materials, Nanowires, Materials science, Nanoscience and technology, Physics, Electrical and electronic engineering

## Abstract

We report the resistivity of 5 Cu films approximately 65 nm thick, measured between 5 and 290 K, and the transverse magnetoresistance and Hall effect measured at temperatures 5 K < T < 50 K. The mean grain diameters are D = (8.9, 9.8, 20.2, 31.5, 34.7) nm, respectively. The magnetoresistance signal is positive in samples where D > L/2 (where L = 39 nm is the electron mean free path in the bulk at room temperature), and negative in samples where D < L/2. The sample where D = 20.2 nm exhibits a negative magnetoresistance at *B* < 2 Tesla and a positive magnetoresistance at B > 3 Tesla. A negative magnetoresistance in Cu films has been considered evidence of charge transport involving weak Anderson localization. These experiments reveal that electron scattering by disordered grain boundaries found along L leads to weak Anderson localization, confirming the localization phenomenon predicted by the quantum theory of resistivity of nanometric metallic connectors. Anderson localization becomes a severe obstacle for the successful development of the circuit miniaturization effort pursued by the electronic industry, for it leads to a steep rise in the resistivity of nanometric metallic connectors with decreasing wire dimensions (D < L/2) employed in the design of Integrated Circuits.

## Introduction

Gordon Moore, cofounder of INTEL, proposed several decades ago an empirical relation (intended to describe advances and the evolution of the circuit miniaturization effort pursued by the electronic industry at the time), that became known as “Moore´s law”. This “law” stated that the number of transistors per unit area contained in a Si wafer doubled approximately every 24 months. However, the resistivity of Cu interconnects has been observed to increase with shrinking dimensions. This fact prompted INTEL in 2016 to announce that “Moore’s law” is coming to a halt. Although the increase in resistivity with shrinking wire dimensions seems an accepted fact, the reasons why such an increase takes place remain unknown; this has become a major obstacle that must be overcome to warrant the success of the continuing effort of circuit miniaturization. According to the road map provided by the IDRS, interconnects are expected to reach widths on the range of 10 to 20 nm within the next decade^1^. Therefore, elucidating and understanding why such an increase takes place has become a central problem within the electronic industry.

We published the first quantum theory of resistivity of nanometric metallic structures arising from electron scattering by grain boundaries and by rough surfaces^2,3^, that allows an estimation of the resistivity of nanometric metallic wires of rectangular cross section in terms of the reflection coefficient R that describes electron scattering *from a single grain boundary*, and in terms of the statistical parameters that describe both the roughness of the surfaces limiting the wire as well as the positional disorder of the grain boundaries within the wire; these statistical parameters are directly measurable on the metallic specimen. This quantum theory (when applied to Cu wires of rectangular cross section)—employing no adjustable parameters—provides the first explanation for the increase in resistivity of about an order of magnitude with shrinking lateral dimensions measured in these wires at room temperature: *Such increase in resistivity is to be expected, for it arises from size effects* (from electron scattering by the rough surfaces limiting the wires as well as by electron scattering from disordered grain boundaries found along an electron mean free path in the bulk) ^4^.

A peculiar feature of this quantum theory, is that it predicts that when the mean grain diameter *D* characterizing the grain size distribution in the nanometric metallic connector is appreciably smaller than L(T), the electron mean free path in the bulk at temperature T, then electron scattering by successive disordered grain boundaries found along a mean free path becomes dominant, and contributes significantly to the increase in resistivity with shrinking wire dimensions, leading to weak Anderson localization. *A distinctive feature of weak Anderson localization is that it gives rise to a negative transverse magnetoresistance.*

In previous research work we measured the magnetoresistance as well as the Hall voltage and the Hall constant on a family of thin gold films deposited onto cleaved mica substrates^5–9^. Most of this work dealt with the magnetoresistance measured on gold films deposited onto mica substrates where the samples were made out of columnar grains extending from the top to the bottom surface of the film^5–9^; only in Ref.^10^ we measured these transport coefficients (magnetoresistance and Hall voltage) in samples made out of both columnar grains as well as samples made out of grains whose typical diameter *D* was smaller than the film thickness t. The measurement of the Hall voltage in these gold films provides some strong empirical evidence that allows elucidating whether charge transport across the sample is controlled by electron-surface scattering (as indicated by a Hall mobility that increases linearly with film thickness t in samples made out of columnar grains), or by electron-grain boundary scattering (as indicated by a Hall mobility that increases linearly with the grain diameter *D* making up the samples when *D* < t). The measurement of the Hall effect in gold samples made out of columnar grains, can also be used to measure nondestructively, the thickness t of the sample, by measuring its Hall voltage^7^. Based upon these results we decided to measure the Hall voltage, the transverse magnetoresistance and the Hall constant in thin Cu films deposited onto cleaved mica substrates varying the mean grain diameter *D* making up the samples. In this paper we report such measurements.

The content of the paper is organized as follows: In Sect. 2 we describe briefly the experimental method. In Sect. 3 we present the results regarding the resistivity of the samples measured between 5 and 290 K, as well as the transverse magnetoresistance, the Hall voltage, the Hall mobility and the Hall constant measured between 5 and 50 K. In Sect. 4 we present the theory used to describe the negative magnetoresistance, that has been considered the “fingerprint” of weak electron localization in thin films. In Sect. 5 we present a discussion of the results.

## Experimental

**Figure 1:**
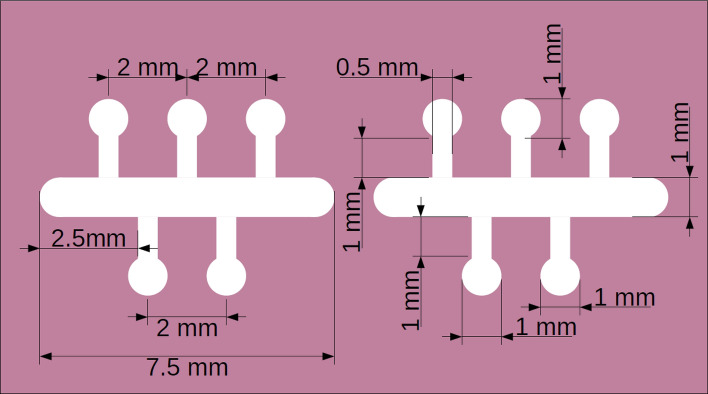
Evaporation mask used to deposit Cu films onto freshly cleaved mica.

The experimental set up used to prepare a family of Cu films approximately 65 nm thick evaporated onto cleaved mica substrates employing Cu 99.9999% pure as a starting material, using a UHV evaporation station, has been briefly described in Sect. 3 of Ref. 4, and is schematically displayed in Fig. 2 of Ref. 4. Changing the substrate temperature from -190 °C to some 35 °C, we were able to control the mean grain diameter *D* making up the samples from some 9 nm to about 35 nm. Following results published by Zarate et al.^11^, the Cu films were covered with a 3 nm thick TiO film at room temperature to passivate the samples before transferring them to a cryostat, in order to perform transport measurements. The mask employed to evaporate the Cu films onto freshly cleaved mica, is displayed in Fig. 1.

Transport measurements were performed by the four-probe method using a Mini Cryogen Free system from Cryogenic Ltd. We measured the temperature dependence of the resistivity for the entire set of samples between 5 and 290 K, as well as the transverse magnetoresistance and the Hall effect between 5 and 50 K, the results are reported below.

## Results

We prepared a family of 5 Cu films deposited on mica, whose characteristics are displayed in Table 1.

**Table 1:** Morphological parameters of the Cu films deposited onto mica substrates. *t* is the film thickness. The histogram describing the grain size distribution of each sample (measured with an AFM), was fitted to a Gaussian distribution having an average grain diameter D and a standard deviation $\sigma$_D ,_ using a set of images of over 1000 grains on samples S1, S2, S4 and S5, and a set of images of 655 grains on S3. R(5) is the resistance of the sample measured at 5 K; R(290) is the resistance of the sample measured at 290 K; ${l}_{\phi }$ is the phase coherence length obtained from fits using the HLN model.

Sample	*t*	*D*	$\sigma$_D_	R(5)	R(290)	${l}_{\phi }$
	[nm]	[nm]	[nm]	[$\Omega$]	[$\Omega$]	[nm]
S1	67.6	8.9	2.6	3.32	4.27	154
S2	65.4	9.8	2.5	2.91	3.79	127
S3	63.2	20.2	5.7	1.82	2.65	109
S4	69.5	31.5	6.6	0.424	1.21	–
S5	65.3	34.7	8.5	0.397	1.17	–

### Temperature dependence of the resistivity

**Figure 2:**
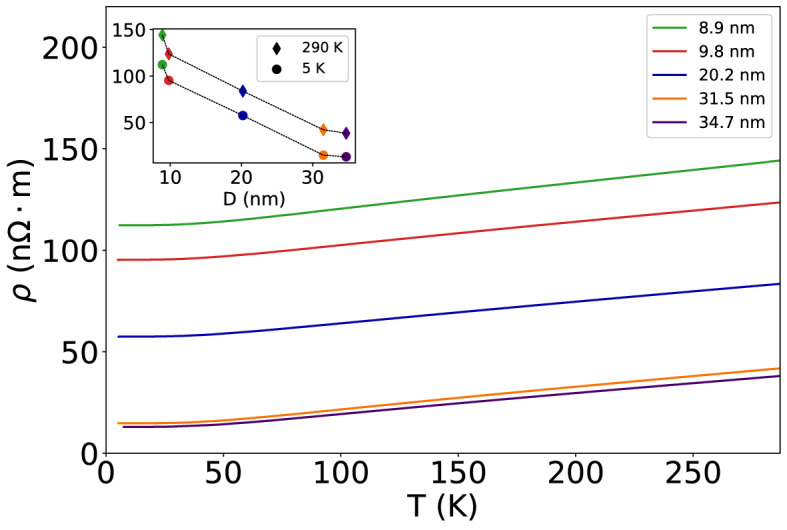
Temperature dependence of the resistivity of the samples measured between 5 and 290 K. In the inset is displayed the resistivity of the samples measured at 5 K and at 290 K, plotted as a function of the mean grain diameter *D*.

The temperature dependence of the resistivity of different samples is displayed in Fig. 2, and in the inset the resistivity of the samples measured at 5 K and 290 K is plotted as a function of the average grain size *D*.

### Transverse magnetoresistance

Let us consider a film oriented along the (*x, y*) plane, with a magnetic field of strength *B* oriented along *z*. The magnetoresistance at temperature *T* is defined as the fractional change in resistivity $\rho \left(B,T\right)$ induced by the presence of the magnetic field *B*

$$\frac{\Delta \rho (B,T)}{\rho (0,T)}=\frac{\rho \left(B,T\right)-\rho (0,T)}{\rho (0,T)}$$

where $\rho \left(B,T\right)$ is the resistivity measured at temperature *T* under an applied magnetic field of strength *B*.

**Figure 3:**
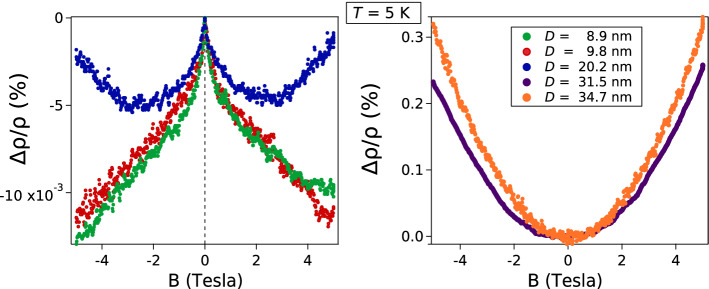
Transverse magnetoresistance of the samples measured at 5 K.

**Figure 4:**
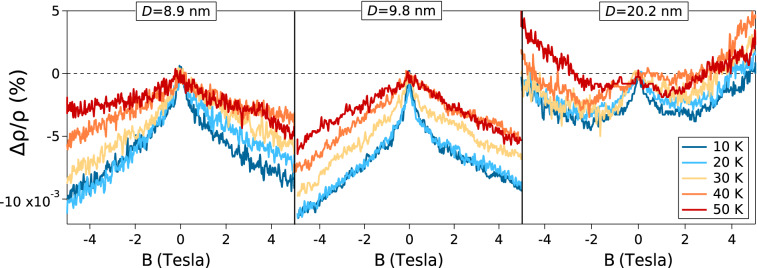
Temperature dependence of the magnetoresistance of the samples for *D* = 8.9 nm, 9.8 nm and 20.2 nm.

The transverse magnetoresistance of the samples measured at 5 K is displayed in Fig. 3, and the magnetoresistance plotted as a function of temperature is displayed in Fig. 4.

### Hall mobility

Carriers moving under the presence of an electric field $\vec{E}$ and a magnetic field $\vec{B}$ experience a Lorentz force that results in an average velocity

$$ \left\langle {\vec{v}} \right\rangle = \mu_{D} \vec{E} + \mu_{D} \mu_{H} \vec{E} \times \vec{B} $$

where ${\mu }_{D}$ is the drift mobility and ${\mu }_{H}$ is the Hall mobility. Under an applied electric field $\vec{E}$ = (E_x_, E_y_, 0) and $\vec{B}$ = (0, 0, *B*), we have $\left( {\left\langle {v_{x} } \right\rangle ,\left\langle {v_{y} } \right\rangle ,\left\langle {v_{z} } \right\rangle } \right) = \left( {E_{x} + \mu_{H} E_{y} B,E_{y}  - \mu_{H} E_{x} B,0} \right)$. The Hall field ${E}_{H}$ is defined as the transverse field needed to cancel out the transverse component of the drift velocity $\left\langle {v_{y} } \right\rangle = 0$ ; it is given by ${E}_{H}$= ${\mu }_{H}{E}_{x}B$. The Hall tangent is defined by $\mathrm{tan}{\theta }_{H}=\frac{{E}_{H}}{{E}_{x}}={\mu }_{H}B$ and the Hall mobility is defined by ${\mu }_{H}=\frac{\partial \mathrm{tan}{\theta }_{H}}{\partial B}$

**Figure 5:**
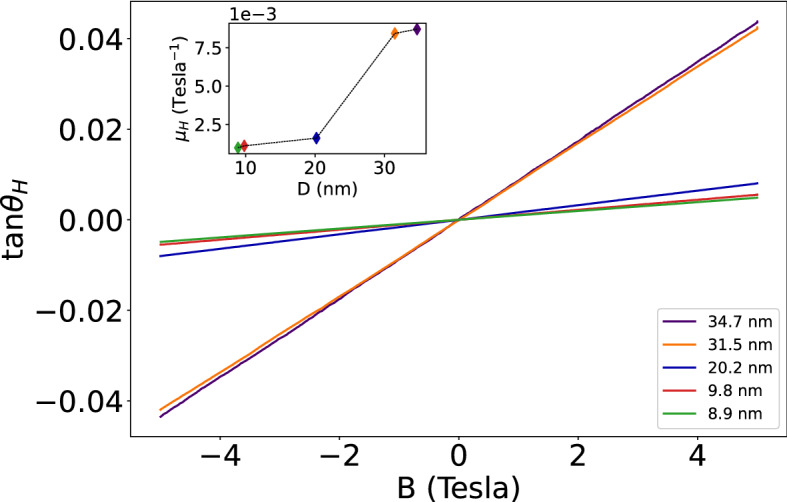
The Hall tangent measured on different samples at 5 K, plotted as a function of magnetic field strength *B*. In the inset is plotted the Hall mobility measured on different samples at 5 K, as a function of the mean grain diameter.

**Figure 6:**
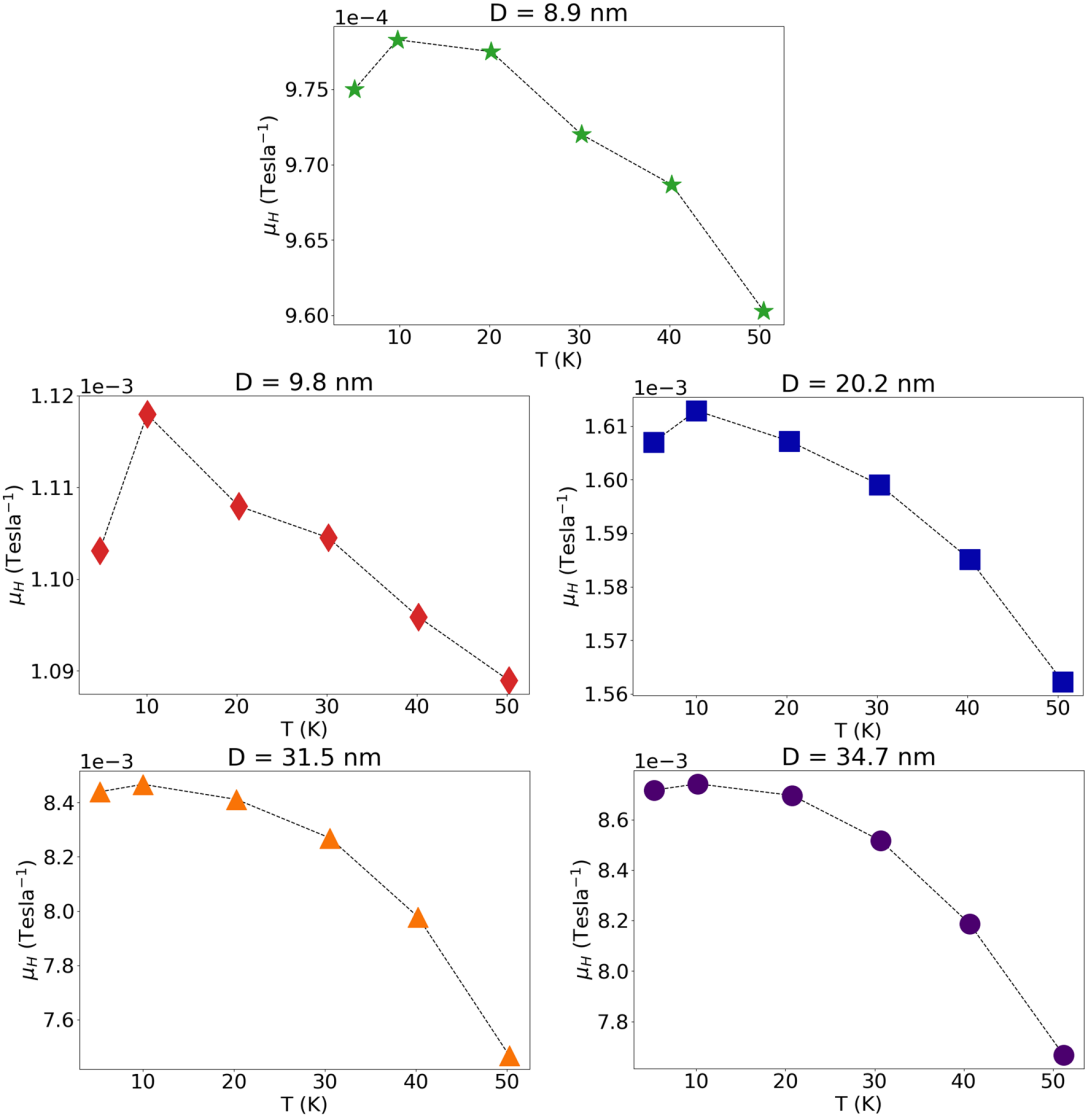
Temperature dependence of the Hall mobility for all samples.

The Hall tangent measured for the entire set of samples at 5 K is displayed in Fig. 5, and in the inset the Hall mobility measured at 5 K is plotted as a function of the average grain diameter *D*. The temperature dependence of the Hall mobility of different samples is displayed in Fig. 6.

### Hall constant

**Figure 7:**
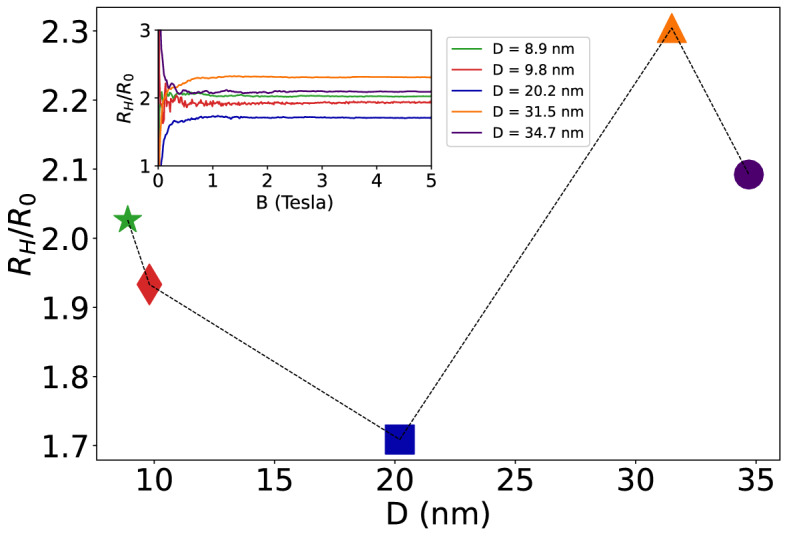
The normalized Hall constant of the samples measured at 5 K plotted as a function of the average grain diameter *D*, computed from ${R}_{H}=\rho {\mu }_{H}$. In the inset we plot the magnetic field dependence of the normalized Hall constant measured at 5 K, computed from ${R}_{H}={R}_{0}\frac{\mathrm{tan}{\theta }_{H}}{B}$.

**Figure 8:**
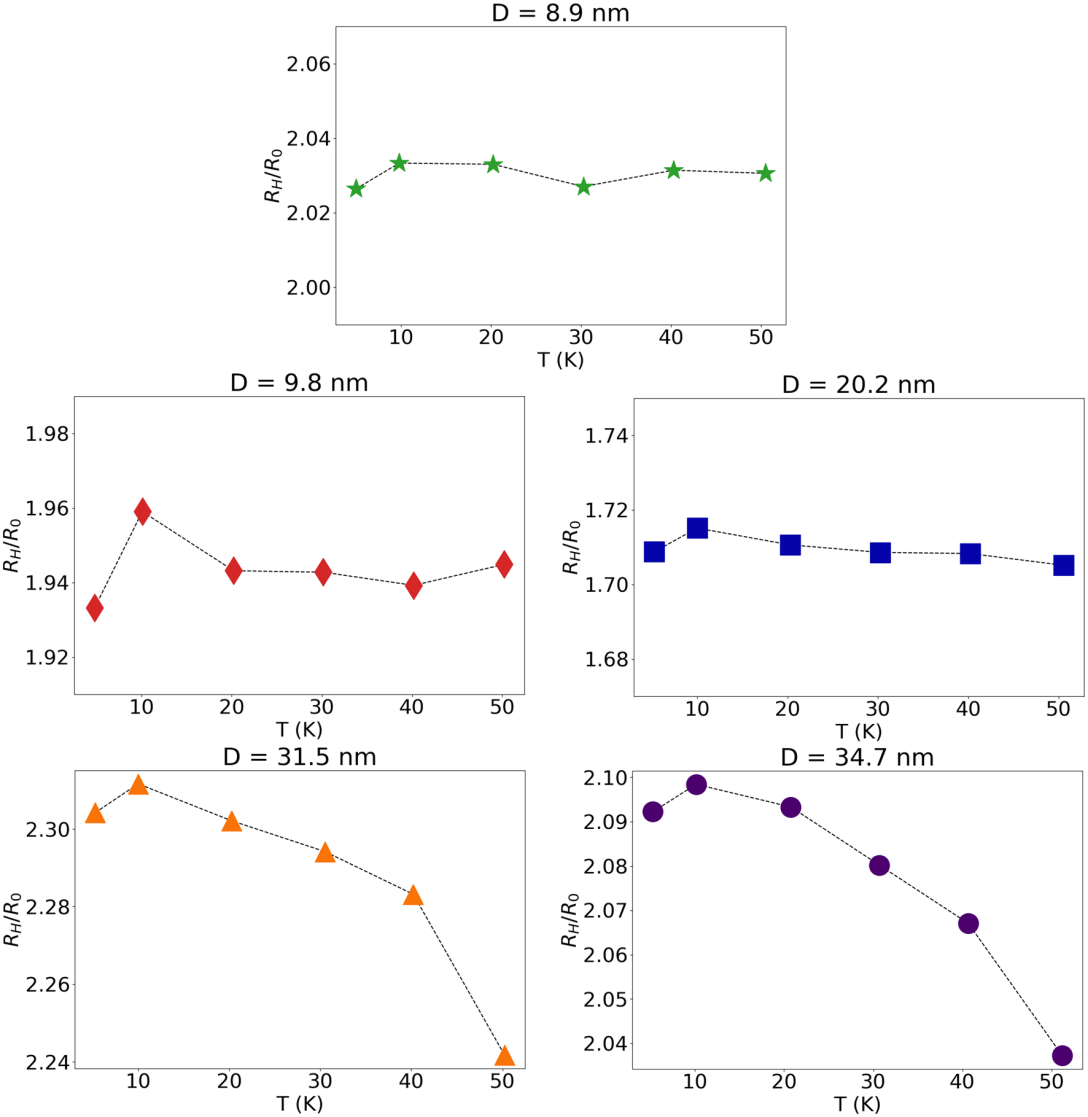
Temperature dependence of the normalized Hall constant for different samples measured at different temperatures, computed from ${R}_{H}=\rho {\mu }_{H}$.

The Hall constant is defined as

$${R}_{H}=\frac{{E}_{H}}{\sigma {E}_{x}B}=-\frac{1}{ne}\frac{{\mu }_{H}}{{\mu }_{D}}=\frac{{R}_{0}}{{\mu }_{D}}\frac{\mathrm{tan}{\theta }_{H}}{B}=\rho {\mu }_{H}={R}_{0}r$$

where ${R}_{0}=-\frac{1}{ne}$ represents the Hall constant corresponding to the electron gas in Cu; ${R}_{H}/{R}_{0}$ is the normalized Hall constant. Here *e* is the electron charge, *n* the electron density, and r is the mobility ratio ${[r=\mu }_{H}/{\mu }_{D}]$. The normalized Hall constant at 5 K plotted as a function of mean grain diameter *D* is displayed in Fig. 7; in the inset we plot the magnetic field dependence of the normalized Hall constant measured at 5 K. The temperature dependence of the normalized Hall constant is plotted in Fig. 8.

## Theory

The theory of quantum corrections to the conductivity arising from the spin orbit interaction in a two-dimensional system containing a random potential leading to a negative magnetoresistance was developed in the early eighties^12,13^. The theory has been applied to explain the negative magnetoresistance in thin Cu films reported by several authors^14–18^.

The basic result is that obtained by Hikami, Larkin and Nagaoka (HLN)^12^, where in absence of magnetic impurities and under a weak spin–orbit interaction, the magneto-conductivity ${\Delta \sigma }_{\perp }$ is [Eq. (18) in Ref^12^]

1 $${\Delta \sigma }_{\perp }=-\frac{\alpha {e}^{2}}{2{\pi }^{2}\hslash }\left[\mathrm{ln}\left(\frac{{B}_{\phi }}{B}\right)-\psi \left(\frac{1}{2}+\frac{{B}_{\phi }}{B}\right)\right]$$

Here $B$ is the applied magnetic field, $e$ the elementary electron charge, $\alpha$ a scaling factor and $\psi$the digamma function. The characteristic magnetic field ${B}_{\phi }$ is defined by ${B}_{\phi }=\frac{\hslash }{4e{l}_{\phi }^{2}}$, where ${l}_{\phi }$ corresponds to the phase coherence length.

## Discussion

It seems interesting to note that when the mean grain diameter *D* decreases from 34.7 nm to 8.9 nm, the resistivity at 5 K increases by nearly one order of magnitude, and it increases monotonically with increasing temperature T. The Hall mobility ${\mu }_{H}$ at 5 K also increases by nearly one order of magnitude with increasing *D* from 8.9 nm to 34.7 nm, and it decreases with increasing temperature. This is in sharp contrast to the normalized Hall constant R_H_/R_0_ that exhibits a rather mild dependence both on T and on D, and turns out to be independent of the strength of the magnetic field B.

This sharp distinction between the grain size dependence of the resistivity and Hall mobility on the one hand, and the Hall constant on the other, is reminiscent of the classical theory of mobility developed in crystalline semiconductors characterized by a spherical Fermi surface, employing solutions of the Boltzmann Transport Equation within the relaxation time approximation $\tau$($\varepsilon$). The mobility ratio turns out to be r = $\upmu$_H_/$\upmu$_D_ =  < $\tau$($\varepsilon$)^2^ > / < $\tau$($\varepsilon$) > ^2^, where the bracket indicates the average of the energy-dependent relaxation time $\tau$($\varepsilon$) employing a weighting function involving the equilibrium electron distribution function f_0_($\varepsilon$) ^7,19^.

If we were to apply these arguments to a metallic specimen endowed with a spherical Fermi surface (rather than to a semiconductor), since the carriers involved in charge transport are those located on the Fermi sphere, we would arrive at the conclusion that r =  < $\tau$($\varepsilon$)^2^ > / < $\tau$($\varepsilon$) > ^2^, hence r = 1 and R_H_ = R_0_. However, Cu is characterized by a Fermi surface that is not a perfect sphere, but consists instead of a sphere plus some necks that bulge out in the < 1,1,1 > , < −1,1,1 > , < 1,-1,1 > and < 1,1,-1 > directions. The experimental results displayed in Figs. 7 and 8 indicate that the normalized Hall constant is roughly independent of B and it exhibits a mild temperature dependence (within the range 1 Tesla < B < 5 Tesla, 5 K < T < 50 K), despite the fact that the charge transport process involves several microscopic electron scattering mechanisms (some of which are temperature-dependent) that are characterized by quite different strengths on the different samples; this point will be revisited below.

However, the most interesting and intriguing result of measuring these magneto transport coefficients, is the remarkable change of sign of the magnetoresistance measured at 5 K displayed in Fig. 3, that is positive for the samples made up of grains where the average diameter is D = 31.5 nm and 34.7 nm, and is negative on the samples where D = 8.9 nm and 9.8 nm. In the sample where D = 20.2 nm, there is a mixed behavior, a magnetoresistance that is initially negative at small magnetic fields *B* < 2 T and then becomes positive as the strength of the magnetic field grows larger than 3 T.

A positive magnetoresistance means that the resistivity increases upon the application of a magnetic field B perpendicular to the film. A negative magnetoresistance, however, means that *the resistivity decreases (and hence the conductivity increases) upon applying a magnetic field.* Such increase in conductivity has been considered a signature of (weak) Anderson localization, and arises when the destructive interference between the wave packets representing the charge carriers interacting with several disordered scattering centers is suppressed because the magnetic field induces a phase factor that *reduces the amount of destructive interference* between these two wave packets [one traveling in the forward and the other traveling in the backwards (reverse) order], hence *the conductivity increases*^12^. Reports of weak localization in thin Cu films have already been published^13–18^. Nevertheless, in the films reported in the present work, the negative magnetoresistance is attributed to electron scattering that becomes dominant as the average size D of the grains making up the sample becomes appreciably smaller than the electronic mean free path L in the bulk, say D < L/2.

**Figure 9:**
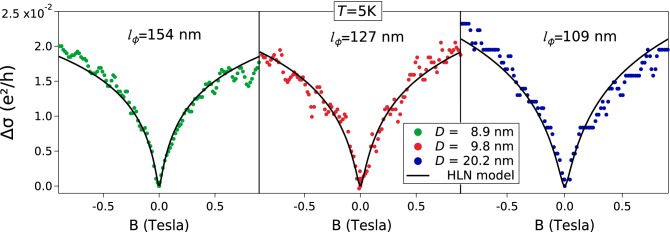
Magnetoconductance curves at 5 K as a function of the applied magnetic field B. The solid lines represent the fitted curves using the HLN model (Ref.^12^), Eq. (1). For the fitted curves we obtained ${l}_{\phi }=$ 154 nm, 127 nm, 109 nm and $\alpha = -$ 511, -604, -747 for the samples S1, S2 and S3, respectively.

We employed the HLN theory to describe the magneto conductivity curves at 5 K by fitting Eq. (1) and using as free parameters the phase coherence length ${l}_{\phi }$ and the scaling factor $\alpha$. In Fig. 9 we display the magneto conductivity curves and the fits as a function of the applied magnetic field in the range from −1 T to 1 T, obtaining ${l}_{\phi }$ for the three samples where weak localization is present. We observe a good agreement between the data and the fits, where the values obtained for ${l}_{\phi }$ are larger than the film thickness, justifying the use of the 2D HLN model.

The new and perhaps the most interesting results reported here, can be summarized as follows: When the mean grain diameter *D* of grains making up the Cu films (approximately 65 nm thick, deposited onto cleaved mica substrates) decreases from 34.7 nm to 8.9 nm, then: (i) the resistivity measured at 5 K increases by nearly one order of magnitude; (ii) the Hall mobility at 5 K decreases by about one order of magnitude; (iii) the transverse magnetoresistance is positive when D > L/2 but becomes negative when D < L/2. Here we have considered L = 39 nm to be the electron mean free path set by electron–phonon scattering in a crystalline sample made out of ultra-pure Cu at room temperature. However L is temperature dependent, it will decrease at higher temperatures and will increase at lower temperatures, although at very low temperatures, such increase may be limited by the mean free path set by electron scattering by impurities/point defects (rather than by electron–phonon scattering) that might be present in a metallic specimen. The reason for choosing ½ as a multiplier of L, is because to give rise to localization (when electrons move across a set of disordered grain boundaries found within L), it is required that the electron wave function traveling forwards in time must interfere with the electron wave function traveling backwards, such that the order in which the electron traverses the barriers is reversed; this phenomenon necessitates a minimum of 2 (or more) grain boundaries found within L. Therefore, *for such interference to take place, the carrier has to traverse at least 2 grain boundaries*. This argument seems consistent and coherent with the crossover, with the tendency of the magnetoresistance to decrease/increase as the strength of the magnetic field B is increased below/above 2.5 Tesla in the sample where D = 20.2 nm displayed in Fig. 3.

We note that there are 2 theories available that describe the resistivity of thin metallic films that include the contribution to the resistivity arising from electron-grain boundary scattering: The classical theory of Mayadas and Shatzkes (MS, Ref.^20^), and the quantum theory of resistivity contained in Refs.^2,3^. In the classical MS theory the current density is computed by calculating the average velocity of the carriers using an electron distribution function that is a solution of the Boltzmann Transport Equation, BTE. In the quantum theory, the conductivity is computed by calculating the velocity autocorrelation of the carriers as prescribed by Kubo’s linear response formalism, employing the Green’s function describing the electron gas confined within the metallic specimen.

In the classical MS theory, when the electric field is oriented along x, the grain boundaries are (y, z) planes located at positions x = x_n_ represented by a series of delta function potentials S$\delta$(x-x_n_) of equal strength S that are, initially, equally spaced, that is x_n_ = nD. Compared to the potential describing the other electron scattering mechanisms that give rise to the resistivity of the bulk, the potential V(x) representing the series of grain boundaries may be considered as a perturbation. In fact, MS employed perturbation theory to calculate a first order correction to the energy of the electron gas, using plane waves to represent the electron wave function. The increase in resistivity (of about an order of magnitude) measured at 5 K when D is decreased from 34.7 nm to 8.9 nm, suggests that the effect of electron grain boundary scattering on the resistivity of the samples is rather large and perhaps should not be considered as a perturbation. Since MS used plane waves to represent the electron wave function, the transmission coefficient T_N_ (describing an electron traversing N consecutive grain boundaries found within L when D < L/2), is T_N_ = 1.

However, if the grain boundaries are equally spaced, then they gives rise to a Kronig-Penney (KP) periodic potential that is superposed onto the periodicity of the crystalline lattice, and hence the electron states become Bloch functions that also exhibit the periodicity D of the lattice of grain boundaries. In MS theory, when D < L/2, all of the increase in resistivity (arising from electron-grain boundary scattering) is attributed to the collision between an electron and the very first element (grain boundary) of this KP potential; the collective effect of electrons traversing successive grain boundaries found within L is entirely ignored because T_N_ = 1. The assumption that all of the increase in resistivity arising from electron-grain boundary scattering arises from the collision between the electron and the very first grain boundary belonging to this periodic lattice of grain boundaries, may be considered a conceptual error. This error becomes particularly severe in samples made up of small grains, D < L/2, for the results reported in this paper indicate that under these conditions, electron-grain boundary scattering plays a dominant role in charge transport.

As discussed in Refs. 2 and 3, this (and other) conceptual errors in MS theory have far reaching implications, such as: (a) When MS theory is employed to describe the temperature dependence of the resistivity data measured between 4 and 300 K in a 49 nm thick gold film where D = 11.1 nm—in Au, L = 38 nm at room temperature—a large reflection coefficient R is needed (R of the order of 0.3 to 0.4 or even higher), and the value of R turns out to depend on the choice of the other parameters inserted into the classical theory [Ref.^3^, and Table 2 from Ref^21^]; (b) Consequently, the fitting parameters contained in MS theory have a questionable validity^2,3^; (c) Further exploration regarding parameter fitting using MS model reveals that the resistivity data corresponding to the same sample can be described using a variety of very different fitting parameters contained in the classical theory (Fig. 22 in Ref.^2^). Hence, the MS theory has no predicting power^2,3^; (d) By setting T_N_ = 1, the MS theory severely underestimates the effect of grain boundary disorder and eliminates any possibility of quantum interference between the electron wave function (traversing several consecutive grain boundaries) traveling forwards and the wave function traveling backwards in time.

On the contrary, in the quantum theory, T_N_ ~ (1-R)^N^—where R is the reflection coefficient of an electron colliding with a single grain boundary^4^. The Green’s function used to compute the conductivity in the quantum theory, was built from the solutions of the Schrodinger equation in the KP periodic potential V(x) made out of grain boundaries equally spaced; thus it naturally includes the allowed and forbidden regions on the Fermi sphere induced by this KP periodicity. And when D < L/2, the quantum theory naturally includes contributions to charge transport arising from carriers that traverse several consecutive grain boundaries. Therefore, the quantum theory is the only theory of resistivity available that leads to weak Anderson localization taking place in samples where the grain diameter D is small enough such that the charge carrier finds 2 or more consecutive grain boundaries within an electronic mean free path L.

In the Cu films reported in this work, one may expect a thin oxide layer to be present between the grains. If the oxide layer has any effect on charge transport, then it should induce a significant increase in the reflection coefficient R needed to describe the temperature dependent resistivity data employing the quantum transport theory (an increase in R over and above the reflection coefficient that would be needed to describe electrons reflected at a grain boundary as a consequence of the misalignment in the crystalline orientation across two adjacent grains in the absence of an oxide layer). However, the resistivity of a Cu film approximately 63 nm thick made up of grains where D = 10.5 nm measured between 5 and 290 K reported in Fig. 3 of Ref.^4^, can be accurately described employing the quantum theory and a (single grain boundary) reflection coefficient of R = 0.12. This is quite close to R = 0.11 needed to describe the resistivity of a gold film 49 nm thick made up of grains where D = 11.1 nm measured between 4 and 300 K employing precisely the same theory^2,3,21^. Since these two reflection coefficients (R = 0.11 for the 49 nm thick Au film deposited on mica, R = 0.12 for the 63 nm thick Cu film deposited on mica) are very close to each other—and we certainly do not expect an oxide layer to be present between the grains of the Au film—we conclude that, if an oxide layer was present between the grains of the Cu films reported in this work, then its effect on the charge transport process can be neglected.

There is yet another sharp distinction between the classical and the quantum theory. Although MS theory was published over 5 decades ago, a classical theory (based upon solutions of BTE) has not been used to calculate the conductivity of metallic wires of rectangular cross section. This is in sharp contrast to the quantum theory, where the Green’s function used to calculate the conductivity can be naturally split into a factor g(x) [built out from the solutions of the Schrodinger equation appropriate to the KP potential V(x) that carries the information regarding equally spaced grain boundaries], multiplied by a function f(y, z) that represents the Green’s function describing the electron gas within the sample.

The function f(y, z) can be chosen to describe electrons confined within two planes parallel to (x, y), appropriate to describe electrons confined in a thin film, or electrons confined between two sets of planes parallel to (x, y) and to (x, z), respectively, appropriate to describe electrons confined within a wire of rectangular cross section. The conductivity in the former case is given by Eq. (41) in Ref. 2; the conductivity of the latter case is given by Eq. (42) in Ref. 2. We note that the conductivity of such metallic wires can be computed employing as input the statistical parameters that characterize the roughness of the surfaces limiting the wire as well as the grain size distribution found within the wire. These parameters are no longer adjustable but can, instead, be measured on a metallic specimen employing a scanning probe microscope. Such extension of the theory has already been applied to describe—without adjustable parameters—the resistivity of Cu wires of rectangular cross section measured at room temperature recently published; these wires are intended to be used as interconnects in the design and construction of integrated circuits corresponding to the 2 nm, 3 nm, 5 nm, 7 nm, 10 nm and 14 nm nodes, respectively ^4^.

Summarizing, the results reported in this paper can be considered as the first experimental evidence indicating that the weak Anderson localization predicted by the quantum theory does, indeed, take place, when electrons traverse several consecutive disordered grain boundaries found within an electron mean free path in a Cu film. We underline the fact that such evidence confirming weak Anderson localization, arises from completely independent experiments. We expect that this phenomenon will play a major role in the charge transport process across a set of disordered grain boundaries, giving rise to an increase in the resistivity observed in a nanometric Cu connector.

This, together with the break-down of Moore’s law observed with shrinking wire dimensions correctly predicted by theory^4^, may be considered as a strong evidence that supports and justifies the description of electron motion upon which the theory of resistivity of nanometric metallic connectors recently published was built. The theory includes a quantum description of each of the following microscopic electron scattering mechanisms that give rise to the observed resistivity: (a) scattering by impurities/point defects, (b) scattering by acoustic phonons, (c) scattering by the rough surfaces limiting the sample and (d) scattering by disordered grain boundaries^2,3^. The published “fingerprint” of the latter mechanism—of weak Anderson localization arising from electron scattering by disordered grain boundaries—is unambiguously revealed by the experiments reported here, in samples where the average grain diameter *D* becomes appreciably smaller than the electron mean free path L in the bulk.

The implications for the effort of circuit miniaturization pursued by the electronic industry worldwide are severe, for it turns out that the break-down of Moore’s law observed with shrinking wire dimensions—a huge obstacle that stands on the way of successfully continuing the effort of circuit miniaturization—is a natural phenomena correctly predicted by the quantum description of electron transport employing no adjustable parameters^4^. According to the description of electron motion contained in this quantum transport theory, in nanometric metallic connectors where *D* < L 2, electron scattering by disordered grain boundaries and electron scattering by the rough surfaces that limit the connector will become dominant and will lead to steep increase in resistivity (of at least an order of magnitude) observed upon shrinking the lateral dimensions of the metallic connector (with decreasing *D* < L 2) to a few nm. This is the reason that explains and quantitatively describes the breakdown of Moore’s law.
