# Author Correction: Evidence of weak Anderson localization revealed by the resistivity, transverse magnetoresistance and Hall effect measured on thin Cu films deposited on mica

**DOI:** 10.1038/s41598-021-98759-2

**Published:** 2021-09-17

**Authors:** Eva Díaz, Guillermo Herrera, Simón Oyarzún, Raul C. Munoz

**Affiliations:** 1 Departamento de Física, Facultad de Ciencias Físicas Y Matemáticas, Universidad de Chile Blanco Encalada 2008, Casilla 487-3, 8370449 Santiago, Chile; 2 Departamento de Física, CEDENNA, Universidad de Santiago de Chile, USACH, Av. Ecuador 3493, 9170124 Santiago, Chile

Correction to: *Scientific Reports* 10.1038/s41598-021-97210-w, published online 08 September 2021

The original version of this Article omitted the Acknowledgements section. The Acknowledgements section now reads:

“S.O. would like to thank the financial support from Basal CEDENNA AFB180001.”

The original Article has been corrected.

